# Development of Bivalent mRNA Vaccines against SARS-CoV-2 Variants

**DOI:** 10.3390/vaccines10111807

**Published:** 2022-10-26

**Authors:** Jianglong Li, Qi Liu, Jun Liu, Zihui Fang, Liping Luo, Shuang Li, Yixin Lei, Zhi Li, Jing Jin, Ronglin Xie, Yucai Peng

**Affiliations:** 1 Liverna Therapeutics Inc., Zhuhai 519000, China; 2 Emergency Key Program of Guangzhou Laboratory, Guangzhou 510000, China

**Keywords:** SARS-CoV-2 variants, mRNA vaccine, spike protein, neutralizing antibody

## Abstract

The severe acute respiratory syndrome coronavirus 2 (SARS-CoV-2) has infected billions of individuals and is the cause of the current global coronavirus disease 2019 (COVID-19) pandemic. We previously developed an mRNA vaccine (LVRNA009) based on the S protein of the Wuhan-Hu-1 strain; the phases I and II clinical trials showed that LVRNA009 has a promising safety and immunogenicity profile. In order to counteract the immune escape by SARS-CoV-2 variants of concern, a panel of mRNA vaccines was developed based on the S proteins of the Wuhan-Hu-1, Delta, Omicron BA.1, BA.2, and BA.5 strains, and each vaccine’s protective potency against the virus variants was evaluated. Furthermore, to achieve excellent neutralization against SARS-CoV-2 variants, bivalent vaccines were developed and tested against the variants. We found that the monovalent Wuhan-Hu-1 or the Delta vaccines could induce high level of neutralization antibody and protect animals from the infection of the SARS-CoV-2 Wuhan-Hu-1 or Delta strains, respectively. However, serum samples from mice immunized with monovalent Delta vaccine showed relatively low virus neutralization titers (VNTs) against the pseudotyped virus of the Omicron strains. Serum samples from mice immunized with bivalent Delta/BA.1 vaccine had high VNTs against the pseudotyped Wuhan-Hu-1, Delta, and BA.1 strains but low VNTs against BA.2 and BA.5 (*p* < 0.05). Serum samples from mice immunized with Delta/BA.2 vaccine had high VNTs against the pseudotyped Wuhan-Hu-1, Delta, BA.1 and BA.2 strains but low VNTs against BA.5. Finally, serum samples from mice immunized with Delta/BA.5 vaccine had high VNTs against all the tested pseudotyped SARS-CoV-2 strains including the Wuhan-Hu-1, Delta, and Omicron variants (*p* > 0.05). Therefore, a bivalent mRNA vaccine with Delta/BA.5 combination is promising to provide broad spectrum immunity against all VOCs.

## 1. Introduction

Coronavirus disease 2019 (COVID-19) is caused by the severe acute respiratory syndrome coronavirus 2 (SARS-CoV-2) and has a devastating influence on public health and the economy globally [1,2]. Since the onset of the SARS-CoV-2 pandemic, several variants of concern (VOCs) have emerged, including Alpha (B.1.1.7), Beta (B.1.351), Gamma (P.1), Delta (B.1.617.2), and Omicron (B.1.1.529) [3]. Currently, the SARS-CoV-2 Omicron VOC has replaced the previously dominant virus variants such as Delta. Omicron sub-lineages show great immune evasions from all vaccines on the market and most of the therapeutic drugs. The neutralization activities of the antibodies against the Omicron variants are dramatically decreased [4,5]. The analyses of serum samples from inactivated vaccines revealed that the neutralization antibody titers were significantly lower against Omicron variants, which indicates increased infection risk [6].

Multiple vaccine platforms have been applied to the rapid development of COVID-19 vaccine candidates, including inactivated vaccine, recombinant subunit vaccine, vector-based vaccine, and messenger RNA (mRNA)-based vaccine [7,8,9,10,11,12,13]. Among them, the mRNA vaccine platform has advantages as a quick pandemic-response strategy in light of its flexibility in immunogen design and the potential for rapid and scalable manufacturing, mainly owing to the high yields of in vitro transcription reactions [14,15]. The mRNA-1273 and BNT162b2 are the only two mRNA vaccines market-approved against SARS-CoV-2 [16,17,18,19]. Both contain mRNA encoding the SARS-CoV-2 Spike (S) protein, the viral receptor-binding domain that recognizes and binds to the receptor angiotensin-converting enzyme 2 (ACE2) on host cells [20,21,22,23,24,25]. Investigation on Omicron escape from neutralization by antibodies from South African individuals vaccinated with Pfizer BNT162b2 showed a 22-fold reduction in vaccine-elicited neutralization by the Omicron variant [26]. Decreases in neutralization titers against the Omicron variants were associated with mutations in the spike proteins [4]. Several approaches are used to keep emerging VOC infections under control. Although primary and booster vaccination with the marketed vaccines can prevent severe COVID-19 and death, an effective vaccine against the current dominant variants and ancestral virus is required [6,27].

Previously, we developed an mRNA vaccine (codename LVRNA009), which was qualitatively analyzed and clinically tested (Phase I: ChiCTR2100049349; Phase II: ChiCTR2200057782); the quality analysis showed that the particle size of the mRNA vaccine was kept at ~65 nm, the encapsulation efficiency was over 95%, the pH was 7.3, and the endotoxin was less than 15 EU/dose. Based on the results from clinical trials, LVRNA009 is promising in safety and immunogenicity at doses of 25, 50, and 100 µg among Chinese adults. In this study, lipid-nanoparticle (LNP)-encapsulated mRNA vaccines expressing the S glycoprotein against different SARS-CoV-2 variants were developed. The immunogenicity of the optimized mRNA LNP vaccines was evaluated. Moreover, bivalent mRNA vaccines were developed, and the effects of the bivalent mRNA vaccines on neutralizing antibodies against the SARS-CoV-2 variants were measured. The results suggest that the bivalent vaccine might offer a promising strategy to manage the COVID-19 pandemic in the future.

## 2. Materials and Methods

### 2.1. Vaccine Design and Production

SARS-CoV-2 vaccines were based on the background of the S protein from Wuhan-Hu-1(GenBank: QHD43416.1), Delta (B.1.617.2) (GenBank: UFO06326.1), Omicron BA.1 (GenBank: UOT56373.1), BA.2 (GenBank: UQJ82268.1), and BA.5 (GenBank: USI08509.1). The vaccines were produced based on the Liverna Therapeutics platform (China patent ZL201911042634.2). Briefly, the mRNAs were synthesized using an optimized T7 RNA polymerase-mediated transcription reaction with complete replacement of uridine by N1-methyl-pseudouridine in vitro. The reaction included a DNA template containing the open reading frame flanked by 5′ UTR and 3′ UTR sequences and was terminated by an encoded poly-A tail. In vitro transcribed (IVT) mRNAs were encapsulated in LNPs according to a modified procedure wherein an ethanolic lipid mixture of ionizable cationic lipid, phosphatidylcholine, cholesterol, and polyethylene glycol-lipid was rapidly mixed with an aqueous solution containing the mRNA products. The analytical characterization of the product was conducted, including the determination of particle size and polydispersity, encapsulation, pH, endotoxin, and bioburden.

The morphology of the nanoparticles was analyzed using transmission electron microscopy (TEM). Briefly, approximately 5 µL of nanoseeds was dropped onto carbon-coated 400 mesh copper grids. The sample was made hydrophilic by glow discharge (EmiTech) for 1 min at 25 mV under atmospheric conditions and then stained with 2% uranyl acetate. After air-drying, the nanoparticles could be viewed, and images were captured at an acceleration voltage of 80 kV.

### 2.2. Verification of S Protein Expression

#### 2.2.1. mRNA Transfection

The HEK293 cells were cultured with Dulbecco’s modified eagle medium (DMEM) supplemented with penicillin/streptomycin and 10% fetal bovine serum (Gibco, Grand Island, NY, USA). The cells were transfected with mRNA using the Lipofectamine^®^ MessengerMAX™ Transfection Reagent (Invitrogen, Carlsbad, CA, USA) according to the manufacturer’s protocol. The transfected cells were incubated at 37 °C with 5% CO_2_ for 24 h before cell harvesting.

#### 2.2.2. Vaccine Antigen Detection by Western Blotting

After transfection, the HEK293 cells were collected and lysed. The protein samples were separated by 12% sodium dodecyl sulfate-polyacrylamide gel electrophoresis (SDS-PAGE) and transferred onto polyvinylidene difluoride membranes. After blocking with 5% skimmed milk for 1 h, the membranes were incubated with anti-SARS-CoV-2 S glycoprotein polyclonal antibody (1:1000, Abcam, Cambridge, UK) and β-actin (1:5000, Abcam, Cambridge, UK) at 4 °C overnight. The secondary antibody HRP-conjugated goat anti-rabbit or anti-mouse IgG (1:5000, BBI Life Sciences, Cambridge, UK) was used to incubate the membrane for 1 h at room temperature. The immuno-stained bands were detected with the enhanced chemiluminescence substrate (Perkin–Elmer, Waltham, MA, USA) exposed to ChemiDoc XRS+ system (Bio-Rad, Hercules, CA, USA).

#### 2.2.3. Flow Cytometry Analysis

After transfection, the HEK293 cells were treated with 20 μg biotin-labeled ACE2 (Rxizi biological science, Wuhan, China) for 2 h. The cells were collected and stained with streptavidin-PE (1:100, BD Biosciences, Heidelberg, Germany) to detect cell surface biotin-labeled hACE2 binding to the vaccine antigen. The cells were acquired using a CytoFLEX Flow Cytometer (Beckman Coulter, Indianapolis, IN, USA) and flow cytometry data were analyzed using the FlowJo software (Tree Star, Inc., Ashland, OR, USA).

### 2.3. Immunogenicity Studies

#### 2.3.1. Animal Experiments and Approvals

The BALB/c mice (female, 5–6 weeks old, average weight of 20 g) were purchased from the Hunan SJA Laboratory Animal Co. (Changsha, China) and the Zhuhai BesTest Bio-Tech Co., Ltd. (Zhuhai, China). Syrian hamsters (female, 4–6 weeks old, average weight of 180 g) were purchased from the Wuhan Institute of Biological Products Co., Ltd. (Wuhan, China). Cynomolgus monkeys (male and female, 5–8 years old, average weight of 3.9 kg) were purchased from the HZ-Bio (Guangzhou, China).

For BALB/c mice (*n* = 10/group), the animals were immunized at days 0 and 14 with a dose of 10 μg. Serum samples were collected 21 days after the first immunization to detect the anti-SARS-CoV-2 Virus neutralization titers (VNTs). The immunization procedure was applied for a panel of vaccines, including the monovalent vaccine against Wuhan-Hu-1, Delta, BA.1, BA.2 or BA.5, and the bivalent vaccines against Delta/BA.1, Delta/BA.2 or Delta/BA.5.

For Syrian hamsters (*n* = 6/group), the animals were immunized at day 0 and 14 with doses of 5 μg or 25 μg monovalent Delta vaccine. Serum samples were collected 20 days after the first immunization to detect the anti-SARS-CoV-2 VNTs.

For Cynomolgus monkeys (*n* = 4/group), the animals were immunized at day 0 and 21 with a dose of 50 μg of monovalent Wuhan-Hu-1 or Delta vaccine. Serum samples were collected 35 days after the first immunization to detect the anti-SARS-CoV-2 pseudotyped VNTs.

All experimental procedures with mice, Syrian hamsters, and Cynomolgus monkeys were conducted according to the Guide for the Care and Use of Laboratory Animals. The mouse studies were approved by the Research Ethics Committee of the Guangzhou Medical University (IAC202202010) and Zhuhai BesTest Bio-Tech Co., Ltd., (IAC202201003). The Syrian hamster experiments were conducted in compliance with the Research Ethics Committee of the Wuhan Institute of Virology (WIVA45202201). The Cynomolgus monkey experimental protocols were approved by the Research Ethics Committee of the Guangdong Laboratory Animals Monitoring Institute (IACUC2020101).

#### 2.3.2. SARS-CoV-2 Plaque Reduction Neutralization Test (PRNT)

The PRNTs for SARS-CoV-2 were conducted as previously described [28]. Briefly, serum samples were serially diluted in DMEM and mixed with an equal volume of SARS-CoV-2 containing 80~100 PFU. Mixtures were added to Vero E6 cells in 12-well plates and incubated for 1 h at 37°C in 5% CO_2_. The supernatants were discarded and replaced with DMEM containing 2% FBS and 1% methyl-cellulose for an additional incubation. After 4 days, the cells were fixed using 10% formaldehyde overnight and stained with 0.5% crystal violet for 25 min. Serum dilutions with a plaque reduction of 50% (PRNT_50_) were referred to as VNTs.

#### 2.3.3. SARS-CoV-2 Pseudovirus Neutralization Assay

Lentiviral particles pseudotyped with different S proteins were produced to compare the neutralizing activity in serum induced by mRNA vaccines against SARS-CoV-2 variants, as previously described [29]. Briefly, the SARS-CoV-2 pseudotyped virus neutralization test begins with serial dilutions of the serum samples, which are then mixed with a certain amount (325~1300 TCID_50_/mL) of the pseudotyped virus. The mixture was added to 293T cells and incubated for 24 h, and the amount of pseudotyped virus entering the target cells is calculated by detecting luciferase expression to obtain the sample’s neutralizing antibody content (VNT). Following this protocol, four samples can be detected simultaneously in a 96-well plate. The cell control (CC) with only cells and the virus control (VC) with virus and cells are set up in each plate. When the raw data for control and samples are exported from the luminometer, the half-maximal effective concentration (EC_50_) is calculated according to the Reed–Muench method for the tested samples.

### 2.4. Viral Challenge Study

#### 2.4.1. Viral Infection

For BALB/c mice (*n* = 4/group), two immunizations with Wuhan-Hu-1 vaccine were performed at day 0 and 14. Mice were anesthetized with isoflurane at day 23, and then transduced with Ad5-ACE2 (2.5 × 10^8^ FFU/75 μL) through the nasal cavity [29]. Five days after Ad5-ACE2 transduction, the mice were infected with SARS-CoV-2 (1.0 × 10^5^ PFU, Wuhan-Hu-1 strain). All mice were euthanized on day 5 after infection, and lung tissues were collected for histopathological examination and virus titer detection.

For Syrian hamsters (*n* = 6/group), two immunizations were performed at day 0 and 14. At day 21, hamsters were intranasally infected with SARS-CoV-2 (Delta strain) (1.0 × 10^4^ PFU/100 μL) [30]. Four days after infection, the trachea and lung tissues of Syrian hamsters were collected for histopathological examination and virus titer detection.

#### 2.4.2. Histopathological Examination

Lung tissues were collected and fixed in a 10% formaldehyde solution. The samples were dehydrated through a graded ethanol series, embedded in paraffin, and cut into 4-μm thick sections. After deparaffinization and rehydration, the sections were stained with hematoxylin and eosin (H&E). The images were photographed under a light microscope.

#### 2.4.3. SARS-CoV-2 Plaque Assay for Virus Titer Detection

The virus, lung, or trachea homogenized supernatants were serially diluted in DMEM. Vero E6 cells were seeded in 12-well plates and cultured with DMEM containing 10% fetal bovine serum (FBS) at 37°C in 5% CO_2_. After removing the inocula, the plates were overlaid with 1.2% agarose containing 4% FBS. Two days later, the overlays were removed, and the cells were fixed. Then the plaques were observed using 0.1% crystal violet staining. Virus titers were calculated as plaque-forming units (PFU) or focus-forming units (FFU) per gram of tissue.

### 2.5. Statistical Analysis

The differences between groups were assessed by an ANOVA test using GraphPad Prism 8.0 (GraphPad Software Inc., San Diego, CA, USA), with indicated significance at *p* ≤ 0.05 (* *p* ≤ 0.05; ** *p* ≤ 0.005; *** *p* ≤ 0.0005; ****p ≤ 0.0001). All results were expressed as means ± standard errors of the mean (SEM) and were corrected for multiple comparisons.

## 3. Results

### 3.1. Design and Expression of the mRNA Vaccines against Different SARS-CoV-2 Variants

**Figure 1:**
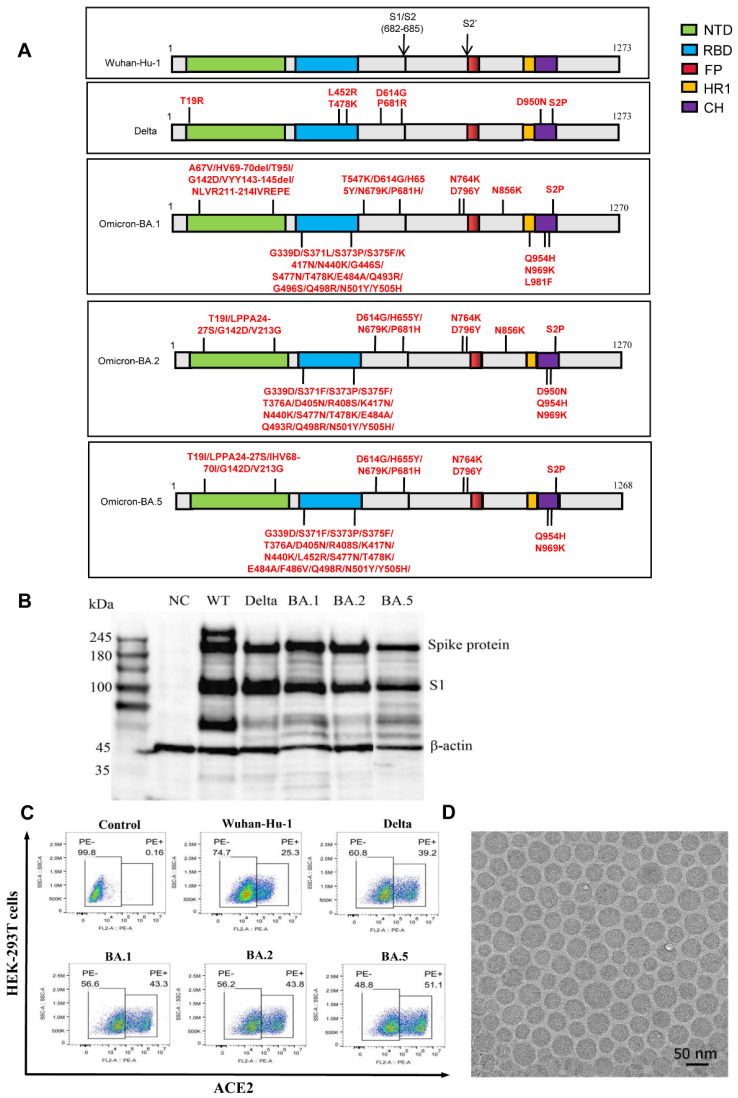
Design and characterization of the mRNA vaccines against different SARS-CoV-2 variants. (**A**) Schematic diagram of the target spike (S) protein-antigen encoded by mRNA vaccines against the Wuhan-Hu-1, Delta, Omicron BA.1, BA.2, and BA.5 variants. The mutation and/or deletion of amino acids are labeled. NTD, N-terminal domain. RBD, receptor-binding domain. FP, fusion peptide. HR1, heptad repeat 1. CH, central helix. (**B**) The expression of the S and S1 proteins in HEK293 cells transfected with IVT mRNA was detected by western blotting. (**C**) HEK293 cells were transfected with IVT mRNA, and hACE2-binding cells were analyzed by flow cytometry. (**D**) Spherical nanoparticles were visualized under a transmission electron microscope. Scale bar, 50 nm.

The S protein, the main target for neutralization antibodies, plays a key role in regulating viral attachment and membrane fusion [20]. The S2P mutation (K986P and V987P) was reported to be a key for stabilizing S protein in prefusion conformation and enhance immunogenicity [30]. Various research groups have applied the S2P mutations to their S protein-based antigen design [31]. Our Wuhan-Hu-1 vaccine was developed without S2P in January 2020, when the benefit of S2P mutation was unclear. In the current study, a panel of mRNA constructs encoding the nucleotide sequence-optimized full-length S protein of SARS-CoV-2 variants was developed into mRNA vaccines, along with the original Wuhan-Hu-1 vaccine. As shown in Figure 1A, compared with the Wuhan-Hu-1 construct (which is wild type encoding the same amino acid sequences as that of the S protein in the SARS-CoV-2 reference strain), the Delta, Omicron BA.1, BA.2, and BA.5 constructs incorporated mutation and/or deletion of amino acids, respectively. Western blotting revealed that the Wuhan-Hu-1, Delta, BA.1, BA.2, and BA.5 constructs induced the expression of the S and S1 proteins (Figure 1B), reflecting the full-length and cleaved S proteins, respectively. Moreover, flow cytometry analysis demonstrated that binding of hACE2, the cell receptor of SARS-CoV-2 [20], was high in all vaccine sample groups (Figure 1C). In addition, compared with the Wuhan-Hu-1 construct, all the Delta, BA.1, BA.2, and BA.5 constructs had increasing ability in hACE2 binding (Figure 1C), suggesting that the mutated S protein could have more effect on the binding capacity of hACE2. The mRNAs with the sequences encoding S protein were encapsulated into LNPs to obtain mRNA vaccine candidates against the Wuhan-Hu-1, Delta, BA.1, BA.2, and BA.5 strains. The LNPs were observed using TEM, and the results showed that the spherical nanoparticles exhibited a uniform size distribution (Figure 1D). Therefore, the mRNA-LNP vaccine has a stable particle structure and meets the delivery conditions of mRNA vaccines.

### 3.2. Monovalent Wuhan-Hu-1 and Delta Vaccines Exerted Potent Protection against Corresponding SARS-CoV-2 Strain Challenges

**Figure 2:**
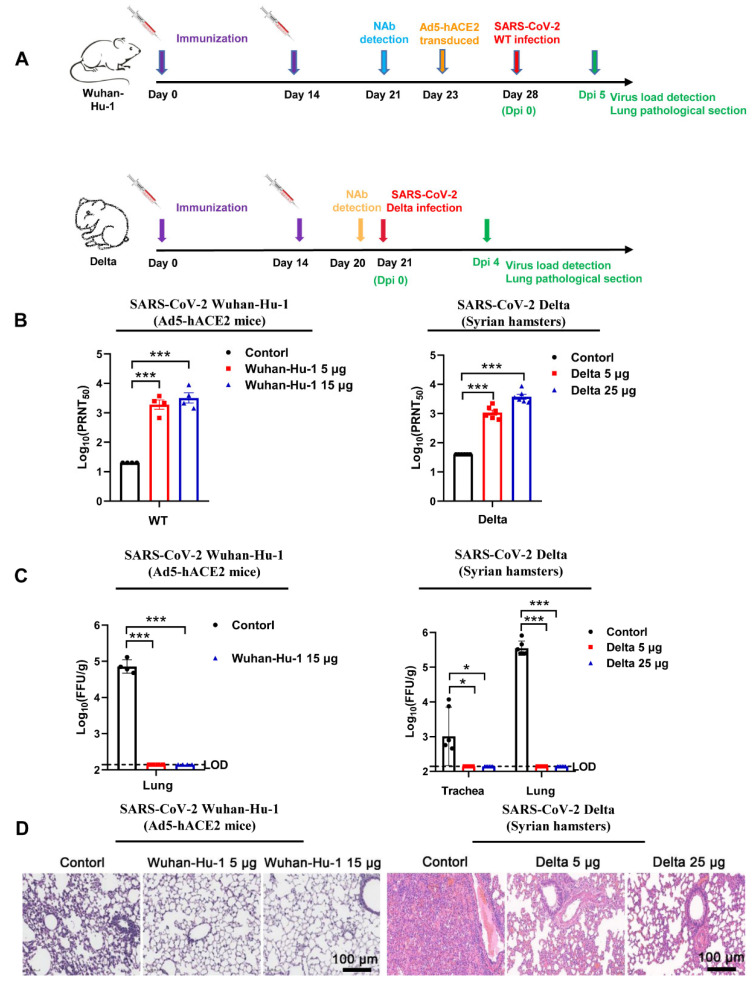
The Wuhan-Hu-1 and Delta vaccines exerted robust protection against SARS-CoV-2. (**A**) Experimental strategy. (**B**) Ad5-hACE2 mice (Wuhan-Hu-1; left panel) or Syrian hamsters (delta; right panel) were immunized twice with low-dose (5 μg/dose) or high-dose (15 or 25 μg/dose) of Wuhan-Hu-1 or Delta vaccine on day 0 and 14. The neutralizing antibody titers against SARS-CoV-2 in animals were tested by PRNT. Ad5-hACE2 mice, *n* = 4. Syrian hamsters, *n* = 6. *** *p* < 0.001 (**C**) Ad5-hACE2 mice or Syrian hamsters in the low- and high-dose groups were challenged with SARS-CoV-2 Wuhan-Hu-1 or Delta. The viral load was analyzed and expressed as FFU per gram of tissue in the lung (Ad5-hACE2 mice; at 4 dpi; left panel) or both lungs and trachea (Syrian hamsters; at 5 dpi; right panel). LOD, Low-limit of Detection. Ad5-hACE2 mice, *n* = 4. Syrian hamsters, *n* = 6. * *p* < 0.05, *** *p* < 0.001 (**D**) H&E staining was used to examine the histopathological changes in lung tissues in Ad5-hACE2 mice (Wuhan-Hu-1; at 4 dpi; left panel) or Syrian hamsters (Delta; at 5 dpi; right panel). Scale bar, 100 µm.

The Ad5-hACE2 mice were immunized twice, on days 0 and 14, using an intramuscular administration of low-dose (5 μg) or high-dose (15 μg) of monovalent Wuhan-Hu-1 strain vaccine, while the negative control group was injected intramuscularly with saline (Figure 2A). On day 21, the VNTs of sera from the monovalent Wuhan-Hu-1 strain vaccine-immunized mice were higher than that of sera from the negative controls (Figure 2B). On day 28 (14 days after the second immunization), the Ad5-hACE2 mice were challenged with 1.0 × 10^5^ PFU of SARS-CoV-2 Wuhan-Hu-1 strain. Plaque assay showed that there was a significant decrease of FFU in lung tissues from the Ad5-hACE2 mice in the low- and high-dose groups in comparison with the negative group (Figure 2C). In addition, severe histopathological changes in lung tissues of the negative control group were observed, while the histopathological changes were much attenuated by the Wuhan-Hu-1 vaccine in all vaccine immunization groups (Figure 2D). These findings suggest that the Wuhan-Hu-1 vaccine promoted inhibition of viral replication in the Ad5-hACE2 mice.

To further confirm the protective efficacy of our vaccines, we used a separate animal model. Syrian hamsters were immunized on days 0 and 14 using an intramuscular administration of low-dose (5 μg) or high-dose (25 μg) of the Delta vaccine, the negative control group was injected with saline (Figure 2A). On day 20, the VNTs of sera from the Delta vaccine-immunized Syrian hamsters were detected to be much higher than from the negative controls (Figure 2B). On day 21 (7 days after the second immunization), the Syrian hamsters were challenged with 1.0 × 10^4^ PFU of B.1.617.2 (Delta variant). Four days later, plaque assay revealed that lower virus titers were detected in the trachea and lung tissues from immunized Syrian hamsters, compared with the samples from the control group (*p* < 0.0005). Similar to observations from the Ad5-hACE2 mice immunized with the monovalent Wuhan-Hu-1 vaccine (Figure 2C), the Syrian hamsters showed a milder degree of lung damage, in all vaccine immunization groups (Figure 2D). These results demonstrated that the Wuhan-Hu-1 or Delta strain vaccines conferred highly efficient protection against SARS-CoV-2 in Ad5-hACE2 mice and Syrian hamsters and the protection potency is dose-dependent.

### 3.3. Profile of Neutralizing Antibodies Produced by Monovalent mRNA Vaccines in Cynomolgus Monkeys

**Figure 3:**
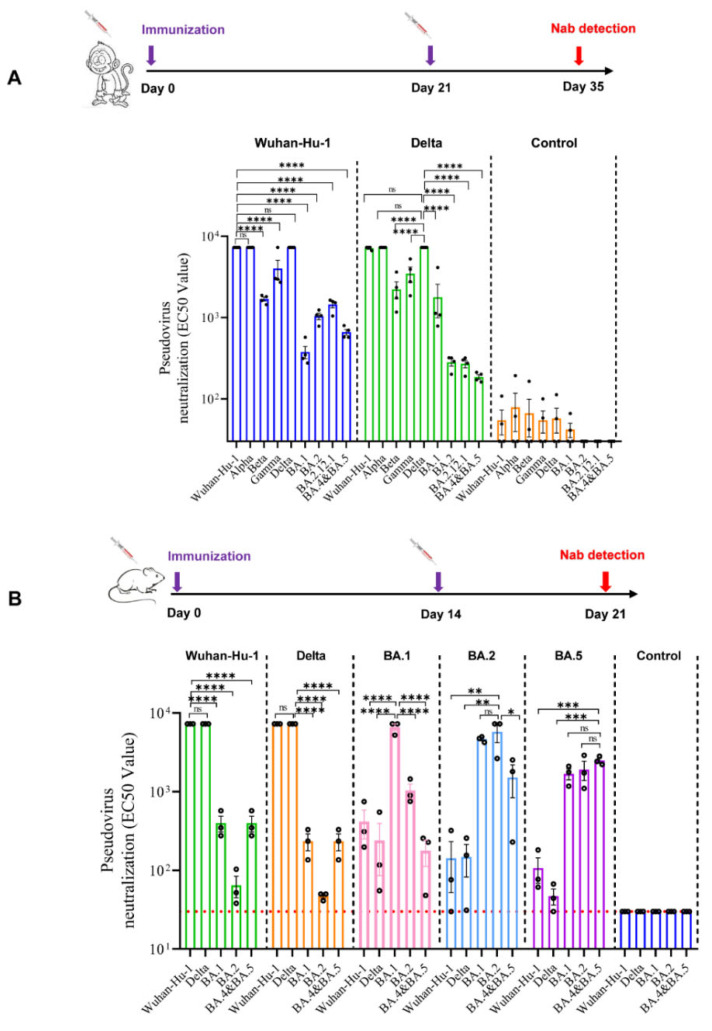
Profile of neutralizing antibodies produced by monovalent mRNA vaccines against SARS-CoV-2 variants in mice and Cynomolgus monkeys. (**A**) Cynomolgus monkeys were immunized twice with monovalent vaccines against Wuhan-Hu-1 strain, Delta strain or PBS on days 0 and 21. The VNTs were measured using a pseudovirus neutralization assay 35 days after the first immunization; *n* = 10. * *p* < 0.05; **** *p* < 0.0001; ns, no significance. (**B**) BALB/c mice were immunized twice with monovalent vaccines against Wuhan-Hu-1, Delta, Omicron BA.1, BA.2, BA.5 strain or PBS on days 0 and 14. The VNTs were measured using a pseudovirus neutralization assay 21 days after the first immunization; *n* = 3. * *p* < 0.05; ** *p* < 0.01; *** *p* < 0.001; **** *p* < 0.0001; ns, no significance.

Cynomolgus monkeys were intramuscularly vaccinated on days 0 and 21 with a single dose of 50 μg (Figure 3A). Comparable studies were conducted on two monovalent vaccines against the Wuhan-Hu-1 strain or the Delta strain. Serum samples were collected on day 35 (14 days after the second immunization) and their pseudotyped VNTs against the SARS-CoV-2 Wuhan-Hu-1, Alpha, Beta, Gamma, Delta, and Omicron variants (BA.1, BA.2, BA.2.12.1, and BA.4&BA.5) were detected (Figure 3A). In the case of the Wuhan-Hu-1 vaccinated serum samples, high VNTs were detected against the Wuhan-Hu-1 and Delta pseudoviruses; there was a slightly reduction in VNTs against the Beta, Gamma pseudoviruses, but significant reduction in VNTs against all the Omicron pseudoviruses (*p* < 0.001) (Figure 3A). For serum samples from the Delta vaccinated animals, a significant reduction in VNTs against all the Omicron pseudoviruses (*p* < 0.001) was also observed (Figure 3A). These findings indicated that the monovalent vaccine against the Wuhan-Hu-1 or the Delta strain may not provide satisfactory protection from the infection of Omicron variants.

### 3.4. Profile of Neutralizing Antibodies Produced by Monovalent mRNA Vaccines in Mice

Mice were intramuscularly immunized twice on days 0 and 14. Serum samples were collected on day 21 (7 days after the second immunization) and their pseudotyped VNTs against the SARS-CoV-2 Wuhan-Hu-1, Delta, and Omicron variants (BA.1, BA.2, and BA.4&BA.5) were detected (Figure 3B). Similar to the results of the monkey studies, the monovalent Wuhan-Hu-1 and Delta vaccines showed relatively high responses against the pseudotyped Wuhan-Hu-1 and the Delta strain but low responses against all of the tested Omicron strains. On the other hand, the monovalent BA.1 vaccine can only induce elevated VNTs against the pseudotyped BA.1, while the monovalent vaccines of BA.2 or BA.5 could induce relatively high level of neutralizing activity against all the tested pseudotyped Omicron strains. These results indicate that antigenic differences between early-stage SARS-CoV-2 strains and the Omicron variants are significant, it is difficult to build up broad spectrum immunity by any monovalent vaccine.

### 3.5. Bivalent Vaccines Produced High Level Neutralizing Antibodies against All SARS-CoV-2 Variants

**Figure 4:**
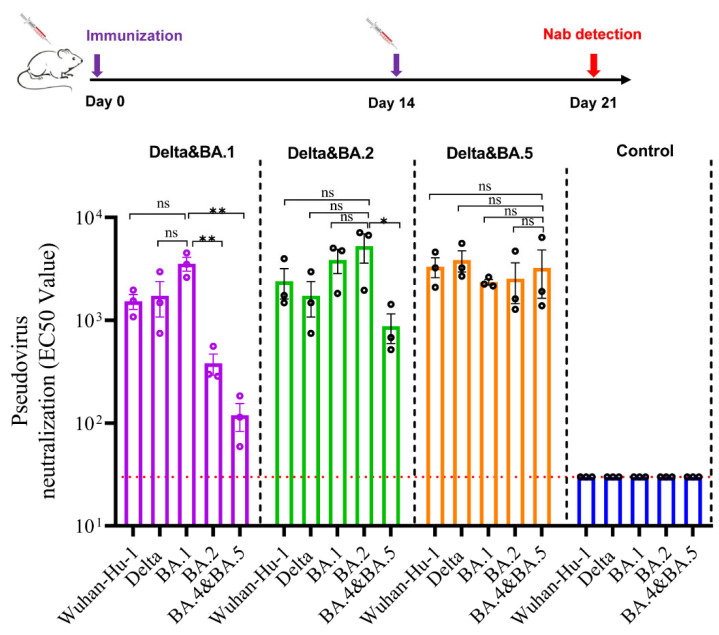
Bivalent vaccine produced high level neutralizing antibodies against the SARS-CoV-2 variants. BALB/c mice were immunized twice with bivalent vaccines (Delta/BA.1, Delta/BA.2, Delta/BA.5) or PBS on day 0 and 14. The VNTs were measured via the pseudovirus neutralization assay on day 21; *n* = 3. * *p* < 0.05; ** *p* < 0.01; ns, no significance.

Based on the studies of monovalent vaccines, we attempted to develop bivalent vaccines to have better coverage against multiple strains. Follow the same immunization procedure as described for mice, serum samples of the bivalent vaccines (Delta/BA.1, Delta/BA.2, and Delta/BA.5) were evaluated against the pseudotyped SARS-CoV-2 Wuhan-Hu-1, Delta, BA.1, BA.2, and BA.4&BA.5 strains (Figure 4). The results demonstrated that serum samples from mice immunized with two doses of the Delta/BA.1 vaccine had high VNTs against the pseudotyped Wuhan-Hu-1, Delta, and BA.1 strain, but low VNTs against BA.2 and BA.5 (*p* < 0.05). The serum samples from mice immunized with the Delta/BA.2 vaccine had high VNTs against the pseudotyped Wuhan-Hu-1, Delta, BA.1 and BA.2 strains, but relatively low VNTs against BA.5. Interestingly, the serum samples from mice immunized with the Delta/BA.5 vaccine had high VNTs against all the tested pseudotyped SARS-CoV-2 strains (*p* > 0.05) (Figure 4). These results indicate that, although bivalent vaccines have differential cross-protective effects against SARS-CoV-2 variants, they demonstrated broader spectrum of immunogenicity. Our data further suggest that the combination of Delta and BA.5 antigens could be an ideal candidate for broadband COVID-19 vaccine development.

## 4. Discussion

To cope with the global pandemic caused by SARS-CoV-2 variants, an effective and broad-spectrum vaccine is urgently needed. In our study, five gene sequences encoding the full-length S proteins of SARS-CoV-2 strains were designed and developed into mRNA vaccines for SARS-CoV-2 mutants (Wuhan-Hu-1, Delta, BA.1, BA.2, and BA.5). Monovalent Wuhan-Hu-1 and Delta vaccines showed potent protection against corresponding SARS-CoV-2 strain challenges in Ad5-hACE2 mice and Syrian hamsters. The immunogenicity potency of the Wuhan-Hu-1 and Delta vaccines was also confirmed by studies with Cynomolgus monkeys, which showed that the mRNA vaccines induced high titers of neutralization antibody against the Wuhan-Hu-1 and Delta strains, but not the Omicron variants. Similar immunogenicity profiles were also observed in studies with mice: Wuhan-Hu-1 or Delta vaccines did not induce high VNTs against Omicron pseudoviruses; BA.1 vaccine only increased VNTs against pseudotyped BA.1; BA.2 or BA.5 vaccine promoted high VNTs against BA.1, BA.2, BA.5, but not against Wuhan-Hu-1 and Delta in mice.

In search of vaccines with broad-spectrum neutralizing activity against a variety of VOCs, we attempted three combinations of monovalent vaccines (Delta/BA.1, Delta/BA.2, and Delta/BA.5) after screening a series of monovalent candidates. When comparing the in vivo immunogenicity of these combinations in BALB/c mice, we found that all three bivalent vaccines could produce neutralizing antibodies against a variety of pseudoviruses, but different combinations had differential immunogenicity profiles. The results showed that the Delta/BA.5 combination could be the most promising bivalent vaccine candidate as it demonstrated broadband and high level of neutralizing antibody activities against all five tested pseudotyped viruses including the Wuhan-hu-1, Delta, and Omicron variants. Recently, Moderna developed a bivalent omicron-containing vaccine mRNA-1273.214 (ancestral + BA.1 VOC) that promotes neutralizing antibody responses against omicron [32]; Pfizer/BioNTech also reported development of bivalent mRNA COVID-19 booster vaccine (ancestral + BA.4/5 VOC) that increases vaccine effectiveness against omicron and elicits the body’s immune response to new emerging variants [33,34]. In comparison, our bivalent vaccine contains Delta and Omicron BA.4/5 variants of SARS-CoV-2. Although pending on clinical validation, our unpublished data indicate that the Delta vaccine has better coverage to VOCs containing D614G mutation than the Wuhan-Hu-1 vaccine. Given the fact that the wildtype Wuhan-Hu-1 strain is no longer prevailing, the combination of Delta and BA.5 should have advantages over Moderna and Pfizer/BioNTech’s approved bivalent vaccines in term of meaningful coverage spectrum.

As a main surface glycoprotein of SARS-CoV-2, the trimeric S protein is ideal for vaccine designation [35,36,37,38]. Our in vitro studies showed that the S proteins expressed by the mRNA constructs of the BA.1, BA.2, and BA.5 variants had stronger binding ability to hACE2 than those of the Wuhan-Hu-1 and Delta variants, which is consistent with reports of increasing infectivity of Omicron variants. At present, the development of broad-spectrum COVID-19 vaccines is focused on strain antigen combination (such as bivalent protein vaccines) or generating new recombinant protein through AI technology targeting the structure of the viral S protein [39]. Some research groups use S protein gene sequence of a certain mutant as the backbone and add immune escape related mutation sites (D614G/L452R/E484A/F486V) to design chimeric mRNA vaccines [3,40]. Up to now, there is no report of a successful development of broad-spectrum recombinant protein based COVID-19 vaccine.

The level of neutralizing antibodies in peripheral blood after vaccination is believed to be a key indicator for evaluating the effectiveness of COVID-19 vaccines [41,42]. In our viral challenge studies with Ad5-hACE2 mice and Syrian hamsters, monovalent vaccines produced high VNTs against the target strains, the animals were well protected from viral infection as indicated by improvement in lung pathology and significant decrease of viral load in lung tissues. The degree of protection was vaccine dose-dependent and highly correlated with levels of serum VNTs. The monovalent Wuhan-Hu-1 vaccine (LVRNA009) was also tested in clinical studies and showed promising safety and immunogenicity profile. As the bivalent Delta/BA.5 vaccine is based on the same technical platform as LVRNA009, its further development shall be constructive and important; we look forward to more clinical data to confirm the preclinical findings presented in this study.

## 5. Conclusions

As SARS-CoV-2 variation causes great immune escape from the first generation of vaccines based on the original Wuhan-Hu-1 strain, a practical strategy is needed for broadband vaccine development. We designed a series of mRNA vaccines based on the full-length S protein of SARS-CoV-2 variants, either monovalent or bivalent. Efficacy studies showed that the monovalent vaccines could well protect animals from challenges by corresponding viral strains. Pseudoviral neutralization assay demonstrated that monovalent vaccine generated antibodies have limited immunogenicity profile against the VOCs; none of the monovalent vaccines could produce satisfactory neutralization antibodies against all VOCs. However, bivalent vaccines demonstrated a much better spectrum, among which the Delta/BA.5 combination is most promising, as it produced high VNTs against all tested VOCs.

## Data Availability

Not applicable.
